# Advances in Microfluidics Techniques for Rapid Detection of Pesticide Residues in Food

**DOI:** 10.3390/foods12152868

**Published:** 2023-07-28

**Authors:** Zhuoao Jiang, Yu Zhuang, Shentian Guo, A. S. M. Muhtasim Fuad Sohan, Binfeng Yin

**Affiliations:** 1 School of Mechanical Engineering, Yangzhou University, Yangzhou 225127, China; jza2422443660@outlook.com (Z.J.); zy1595971369@hotmail.com (Y.Z.); gst3883@163.com (S.G.); 2 Faculty of Engineering, Department of Mechanical Engineering, The University of Adelaide, Adelaide, SA 5000, Australia; asmmuhtasimfuad.sohan@student.adelaide.edu.au

**Keywords:** pesticide residues, microfluidic, rapid detection, food samples

## Abstract

Food safety is a significant issue that affects people worldwide and is tied to their lives and health. The issue of pesticide residues in food is just one of many issues related to food safety, which leave residues in crops and are transferred through the food chain to human consumption. Foods contaminated with pesticide residues pose a serious risk to human health, including carcinogenicity, neurotoxicity, and endocrine disruption. Although traditional methods, including gas chromatography, high-performance liquid chromatography, chromatography, and mass spectrometry, can be used to achieve a quantitative analysis of pesticide residues, the disadvantages of these techniques, such as being time-consuming and costly and requiring specialist staff, limit their application. Therefore, there is a need to develop rapid, effective, and sensitive equipment for the quantitative analysis of pesticide residues in food. Microfluidics is rapidly emerging in a number of fields due to its outstanding strengths. This paper summarizes the application of microfluidic techniques to pyrethroid, carbamate, organochlorine, and organophosphate pesticides, as well as to commercial products. Meanwhile, the study also outlines the development of microfluidics in combination with 3D printing technology and nanomaterials for detecting pesticide residues in food.

## 1. Introduction

Pesticides are crucial in contemporary agriculture because they prevent crop losses from pests. They also protect crop growth and yields. The wide application of new pesticides has improved agricultural production, but the food safety problem caused by them has attracted more and more attention. Pollution caused by pesticides has gradually become a global public health problem [1,2,3,4]. The excessive intake of pesticides seriously harms human health [5,6,7]. Overuse, heavy reliance, and improper processing have left residues in crops and enriched them in the human food chain [8]. The consumption of foods that are high in pesticides can cause endocrine disorders, cancer, and neurological diseases [9,10,11]. The risks posed by pesticide residues are more acute for children and expectant women [12,13]. The entire food business faces a severe challenge due to this focus. The food business and producers are subject to more intense scrutiny and demand to ensure the quality and safety of food due to greater regulatory enforcement and customer awareness. One of their most essential tasks is identifying pesticide residues in food to safeguard people’s lives and health [14]. However, conventional pesticide detection technologies have numerous shortcomings that mean they cannot be used as rapid on-site detection technology [15,16,17].

The primary traditional methods for detecting pesticide residues are gas chromatography, high-performance liquid chromatography, and mass spectrometry [18,19,20,21]. These detection techniques have the advantages of accuracy and sensitivity. However, their sample processing and pretreatment procedure is complicated, time-consuming, expensive, and labor-intensive. As a result, traditional detection technology cannot meet the needs of consumers for the rapid and convenient detection of pesticide residues. Therefore, developing a technology that can rapidly, conveniently, efficiently, and sensitively detect pesticide residues in food is essential. The demand for point-of-care testing for food safety is answered by microfluidics. Microfluidics provides a platform for rapidly detecting trace pesticide residues with a small sample. Combining microfluidic technology with pesticide residue detection devices effectively overcomes the shortages of traditional methods and realizes on-site detection [22,23,24,25,26].

Microfluidics integrates various functional units in submillimeter microchannels for a variety of analytical chemistry operations such as purification [27,28], reaction [29,30], separation [31,32], and detection [33,34]. Microfluidic sensors have the advantages of high throughput, miniaturization, portability, and small reagent consumption [35,36,37,38,39,40,41], which can rapidly obtain more accurate detection results. It is significant for food safety to develop on-site detection technologies and portable equipment [42,43,44]. Microfluidic sensors can identify specific analytes through biomolecules and enhance them into detectable signals [45,46,47,48]. However, to our knowledge, very few reviews currently expand on the application of microfluidics in detecting food pesticide residues [49].

**Scheme 1:**
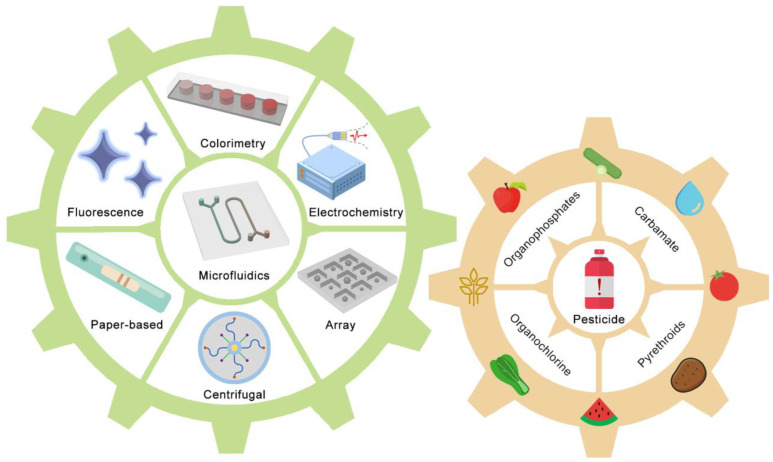
Microfluidic-technology-based schematic diagram for the detection of food pesticide residues.

This article reviews the recent research progress of microfluidics in rapidly detecting food pesticide residues, hoping to provide new ideas for future microfluidics in pesticide detection. It focuses on several microfluidic devices to detect the most commonly used pesticides globally, including pyrethroid, carbamate, organochlorine, and organophosphate pesticides. Different microfluidic tools like paper, arrays, and centrifugation and signal readouts like colorimetry, fluorescence, and electrochemical approaches are used for various food samples, as illustrated in Scheme 1. Future trends and commercial technologies for the on-site detection of pesticide residues are explored.

## 2. Microfluidic Devices for Pesticide Detection

Due to the advantages of microfluidics in point-of-care testing, convenient, rapid, and efficient chemical reactions have become the first choice for detecting food pesticide residues [50,51,52,53,54]. Various microfluidic devices detect food pesticide residues, such as those from organophosphates, carbamates, and pyrethroids [55]. Table 1 lists exemplary microfluidic tools for rapidly detecting pesticide residues in food.

### 2.1. Organophosphates Compounds

Pesticides with an organophosphate chemical as their primary component are known as organophosphate pesticides [62,63]. These insecticides are commonly used in horticulture and agriculture to improve crop yield and quality while controlling various pests and illnesses. Organophosphate pesticides primarily poison pests via acetylcholinesterase inhibition [64,65]. However, the nervous system of people might also be impacted by this [66]. Prolonged or excessive exposure to organophosphate residues may cause neurological symptoms such as headache, dizziness, nausea, vomiting, muscle cramps, neurasthenia, and memory loss [67,68,69]. There are numerous studies on the detection of organophosphate pesticides [70,71,72,73,74,75,76]. Shi and colleagues used phage and horseradish peroxidase to create an eco-friendly and safe electrochemical immunosensor [77]. Li et al. developed a MnO_2_ switch-bridged DNA walker for the ultrasensitive sensing of ChEs activity. It can effectively detect organophosphorus pesticide residues in actual samples [71]. Hurija et al. developed a conjugated polymer and core–shell magnetic nanoparticle-containing biosensor for pesticide analysis [72]. Under optimized conditions, the biosensor in concern revealed a rapid response (5 s), a low detection limit (6.66 × 10^−3^ mM), and high sensitivity (45.01 μA mM^−1^ cm^−2^).

**Figure 1:**
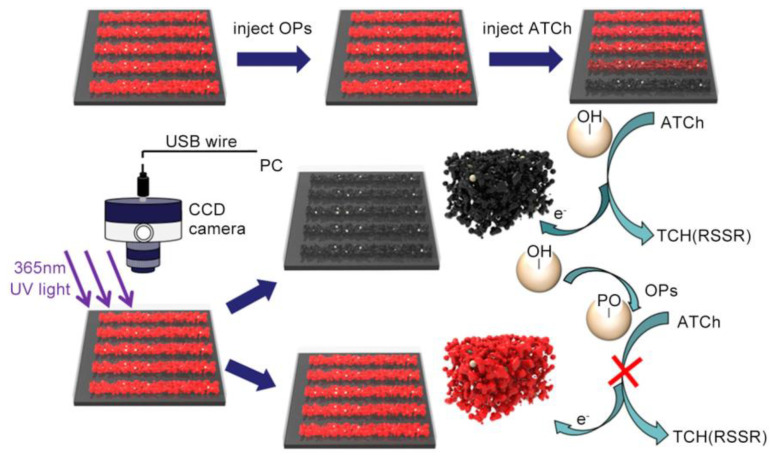
Schematic diagram of organophosphate fluorescence detection based on enzyme-inhibited recovery reaction. (Reprinted/adapted with permission from Ref. [24]. Copyright 2019 *Biosensors & Bioelectronics*).

A microfluidic device based on fluorescence intensity for quick pesticide residue detection in food has higher sensitivity compared to the conventional method [78,79,80,81,82,83,84], and Hu et al. (2019) developed a microfluidic array sensor based on QD-AchE aerogel that can detect organophosphates pesticide residues quickly and with high sensitivity [24]. The principle of using the microfluidic device to detect pesticide residues via fluorescence intensity is shown in Figure 1. Quantum dots’ fluorescence intensity gradually increases with an increase in organophosphate concentration. Since acetylcholine catalyzes the production of thiochotine, organophosphates inhibit its activity and restore the fluorescence intensity of acetylcholine-quenched quantum dots. With detection limits of less than 1.2 pM and a detection range of 10^−5^ M–10^−12^ M, the researchers evaluated three popular organophosphate pesticides, including paraoxon, parathion, and dichlorvos. This further proved that the sensor has high sensitivity and a broad detection range. Additionally, they evaluated that the recovery of organophosphates insecticides achieved 98% using apple samples. However, the instrument is currently based on monochromatic fluorescence to detect organophosphate pesticides, which results in limited detection sensitivity due to the low contrast between red and the background color. In the future, the contrast can be increased by adding various colors to improve the detection sensitivity.

**Figure 2:**
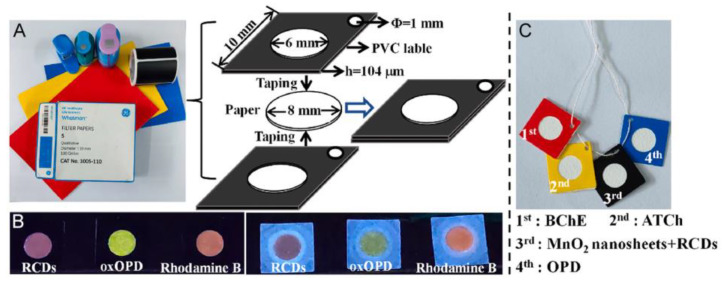
(**A**) Schematic diagram of 2D μPAD. (**B**) Fluorescence detection image. (**C**) Schematic diagram of 3D μPAD. (Reprinted/adapted with permission from Ref. [25]. Copyright 2023 *Biosensors & Bioelectronics*).

Also, based on fluorescence detection, compared with the array microfluidic sensor developed by Hu et al., Tong et al. developed a threaded paper-based microfluidic device [25], as shown in Figure 2 below. Using 3D printing technology and fixed with cotton thread, threaded 3D paper-based microfluidic analytical devices (μPADs) (Figure 2C) included four 2D μPADs (Figure 2A). They created a ratio fluorescence system for organophosphate detection using MnO_2_ nanosheets to oxidize o-phenylenediamine into 2,3-diamino phenazine with yellow-emission fluorescence and the internal filter effect to quench the fluorescence intensity of red emission carbon dots (RCDs). The fluorescence detection image is shown in Figure 2B. They also chose actual samples of spinach and tomatoes, with recovery rates ranging from approximately 94.0% to 106.0% and relative standard deviations (RSDs) under 8.6%. The test results matched those from the HPLCMS test. This technique performs well and is appropriate for accurate field organophosphate identification in real samples. The design diversity of 3D μPAD provides a simple and efficient detection platform for the detection of pesticide samples in complex agricultural samples.

**Figure 3:**
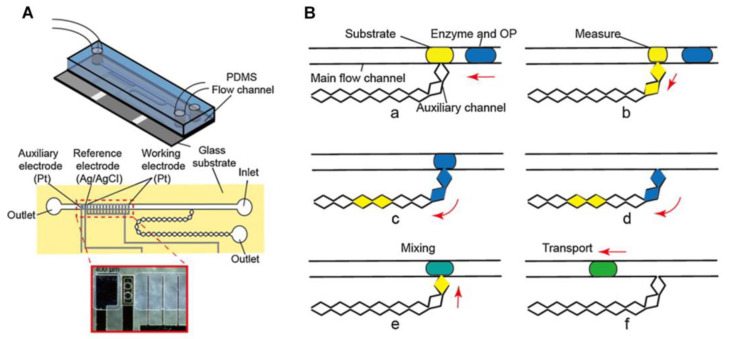
(**A**) Schematic diagram of a microfluidic device. (**B**) Solution mixing procedure diagram. (a) The first step is to introduce the reaction solution into the mainstream channel. (b–d) Measure the volume using the auxiliary runner and discard the main runner section. (e) The two solutions merge in the main channel. (f) Transport the new plug to the sensing area. (Reprinted/adapted with permission from Ref. [56]. Copyright 2014 *Sensors and Actuators B-Chemical*).

Electrochemical technologies [85,86,87,88] are more straightforward and sensitive than fluorescence detection because they directly transform difficult-to-measure chemical parameters into simple-to-measure electrical ones. Common electrochemical identification techniques frequently demand intricate electrode production procedures and expensive detection costs. Yang et al. suggested a method for identifying pesticide residues based on multilayer paper-based microfluidic chips to address this issue [89]. After spraying pesticides on lettuce, the avermectin, phoxim, and dimethoate identification accuracy remained consistent at 93%. A stopper microfluidics-based organophosphate-pesticide-detecting system was created by Wang et al. (2014) [56]. As shown in Figure 3A, the device consists of a glass substrate measuring 11 mm × 33 mm, a Kühler-style inspection thin-film three-electrode system, and a polydimethylsiloxane (PDMS) substrate with a flow channel structure. The instrument employs hydrogen peroxide to oxidize the microelectrode array. Acetylcholinesterase activity changes following the addition of organo-phosphorus pesticides, and the charge change is determined using the Kuhler method. Figure 3B shows the procedure used to process plugs at the T-junction. Finally, the concentration of organophosphate pesticides is measured. The charge resulting from the organophosphate concentration’s logarithm has a linear connection. Malathion’s lower detection limit (LOD) is 33 nM, while the LOD for acephate, methamidophos (MEP), and diazinon are 90 nM. The Kuhler method, which relies on inhibiting acetylcholinesterase, can be carried out with tiny volume stoppers, requiring fewer expensive chemicals. The fast mixing of plugs makes it easier to repeat experiments and take accurate readings.

### 2.2. Carbamate Compounds

Carbamate pesticides are widely used in agriculture and forestry because of their high selectivity, easy decomposition, little residual toxicity, and low toxicity to humans and animals [90,91,92,93]. However, carbamate pesticides with heavy usage in foods spread through the food chain and accumulate in the human body through the digestive system and the skin’s mucous barrier [9,94,95,96]. In various studies, carbamate pesticides have been shown to quickly produce nitroso compounds with nitrite in food (bread, yogurt, cheese, soy sauce, and vinegar), which can substantially harm human health [97,98]. They are also mutagenic, teratogenic, and carcinogenic under acidic circumstances in the stomach [99]. In this case, some researchers have proposed various methods of detecting carbamate [100,101,102,103]. However, the sample handling and pretreatment steps required for these procedures are complex and time-consuming. To solve these problems, desirable methods like fluorescence, colorimetry, and electrochemistry were proposed with high sensitivity and rapidity [104,105,106]. Wu’s group discovered Cu^2+^/Cu^+^ conversion as the electrochemical signal for detecting ethyl carbamate. To achieve the visual detection of carbamate, Chen’s group proposed a fluorescence paper-based sensor to detect carbamate in food [107]. To achieve the automation of detection, Yan’s group designed a multi-signal readout platform for the sensitive monitoring of carbamate pesticides [108]. Currently, these desirable methods still suffer from long-distance transportation and complex environments.

**Figure 4:**
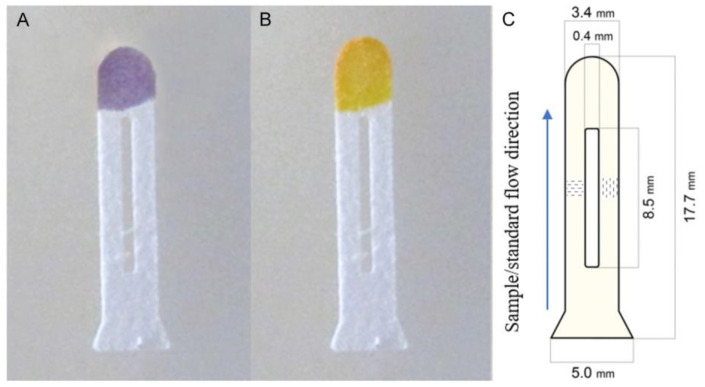
(**A**) Schematic diagram of μPAD when adding pesticides. (**B**) Schematic diagram of μPAD in the absence of pesticides. (**C**) Drawing of design of μPAD. (Reprinted/adapted with permission from Ref. [57]. Copyright 2020 *Talanta*).

Microfluidic devices [109,110] offer a viable solution to achieve the detection of carbamate and overcome the issues associated with complex procedures and transportation. Interestingly, microfluidics widely utilize unitary and multiple signal readouts [111,112,113,114,115]. For instance, based on colorimetry, M.D. Fernández-Ramos (2020) suggests a bioactive microfluidic paper device for pesticide determination in water [57]. The proposed device contains three independent regions: a μPAD at the bottom for sampling, two microchannels separated by deposited acetylcholinesterase and AChCl solutions, and a top μPAD containing a pH indicator for detection. The paper device, working at room temperature, sets the reducing reaction’s rate as an analytical signal to be quantified based on the color of μPAD. Figure 4A,B display two color diagrams to verify carbamate’s existence, where the purple one is deemed as the presence of carbamate, and the yellow one is regarded as the absence of carbamate. A drawing of the design of the whole device is exhibited in Figure 4C to guide the microfluidic chip fabrication. The concentration of carbaryl was determined using calibration curves and found to be 2.00 μg L^−1^ for carbaryl. It ranged from 5.5%, and the detection limit was 2.00 μg L^−1^. The researchers also conducted recovery trials with known concentrations of carbaryl, with an average recovery rate of 97.7%. The device with the capillary holders can be used to conduct many analytical processes, such as sample buffering, sample filtration, etc.

**Figure 5:**
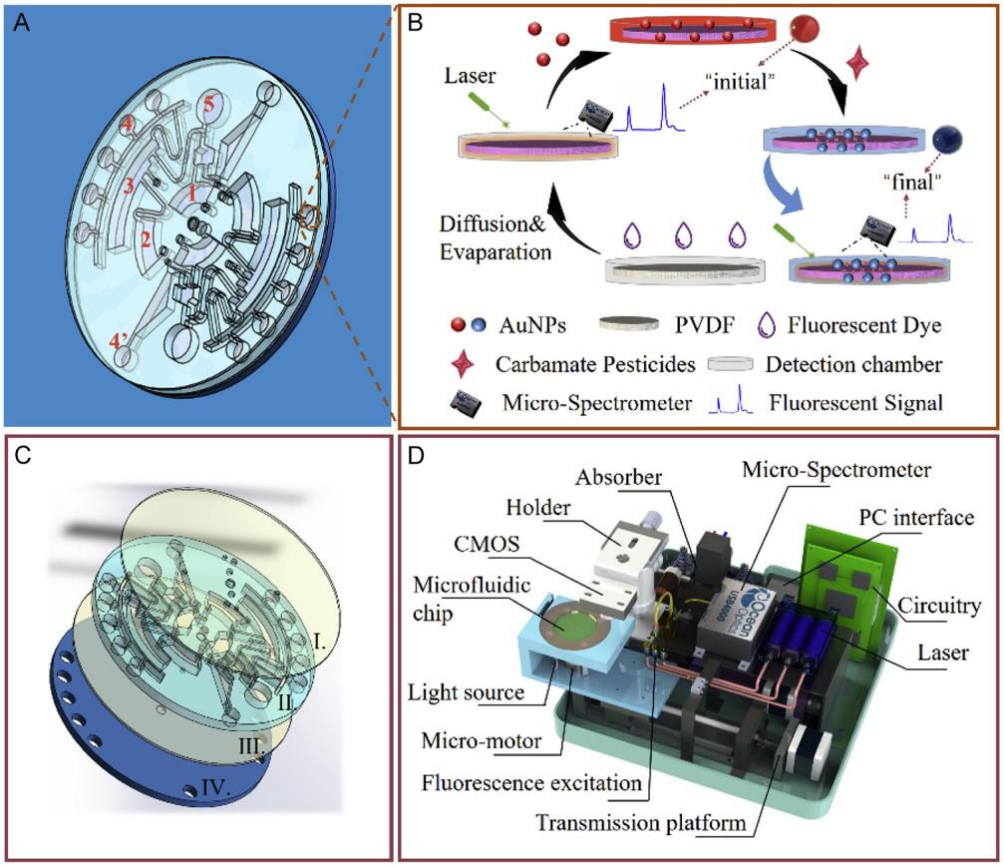
(**A**) Internal structure diagram of a microfluidic chip. (**B**) Schematic diagram of the detection principle. (**C**) Schematic diagram of a 3D-printed, four-layer microfluidic chip. (**D**) Schematic diagram of the device used to inspect the chip. (Reprinted/adapted with permission from Ref. [23]. Copyright 2021 *Sensors and Actuators B-Chemical*).

Meanwhile, multiple readouts are successfully utilized to achieve the detection of carbamate, increasing sensitivity and integration. Zhao et al. (2021) built a portable automatic double-readout detector integrated with a 3D-printed microfluidic nanosensor on the foundation of the colorimetric method [23]. As shown in Figure 5A,C, the chip, containing five chambers and several channel structures, was designed to control the flow and detection of chemical mixtures through centrifugal force [116,117,118]. The device, capturing chromatic aberration and fluorescence spectral images, successfully distinguished six urethane pesticides based on the cross-response mechanism and agglomeration effect of gold nanoparticles (AuNPs) (Figure 5B). It demonstrated high sensitivity and selectivity for urethane pesticides at the ppb level and good recognition ability at low concentrations of 50 ppb–800 ppb. With convenience and integration, the device can also be adapted for environmental monitoring and home testing (Figure 5C).

**Figure 6:**
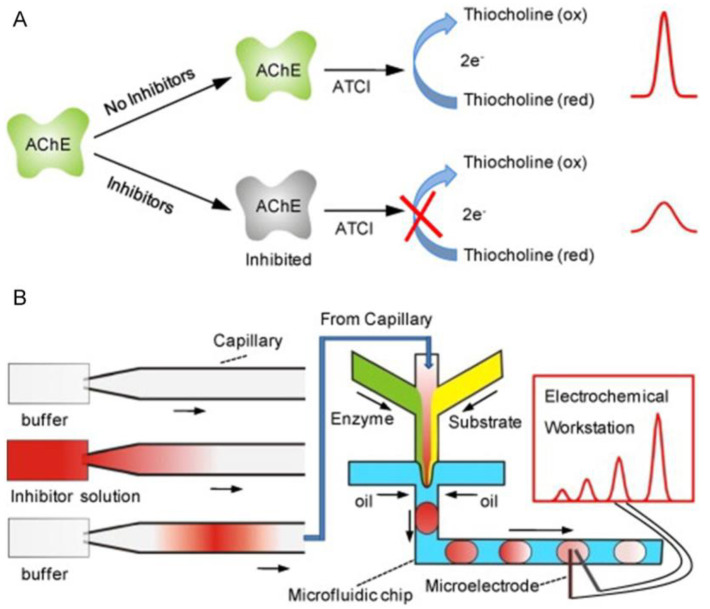
(**A**) Schematic diagram of the detection principle of acetylcholinesterase inhibition based on the electrochemical method. (**B**) Schematic diagram of pesticide testing system. (Reprinted/adapted with permission from Ref. [59]. Copyright 2013 *Analytica Chimica Acta*).

The advantage of employing an electrochemical approach over a fluorescence method is that the analyte of interest does not need to be coupled to a fluorescent reporter or an imaging setup. It simply requires a pair of electrodes, which are highly sensitive and versatile and are easily shrunk and integrated into a microfluidic platform. Based on electrochemical microfluidics, Flavio et al. suggested a method for quickly and accurately detecting phenyl carbamate herbicides in rivers, lakes, and irrigation water samples. This technique significantly enhances the C18-based OMIX microtip approach, which enriches the analyte by a factor of 10, lowers reagent waste, and increases the accuracy of detection results. It can quickly separate and sensitively detect carbamate pesticides in 6 min. Meanwhile, Gu et al. coupled concentration gradient creation and electrochemical detection to fabricate a straightforward and reliable droplet dose-reactive enzyme inhibition microfluidic device [59]. To introduce reagent and construct concentration gradients, a variety of slotted flasks and conical-tip capillaries were implicated in this device (Figure 6A). PDMS, which is integrated with microelectrodes, is used for droplet production and electrochemical detection. The method is based on the enzyme inhibition principle shown in Figure 6B, and it was used to calculate the average semi-inhibitory concentration value of carbaryl with less than 5 μL of total reagent.

### 2.3. Other Pesticides

Organochlorine pesticides are organic compounds containing chlorine in their chemical structure, which are fat-soluble and kill insects by interfering with the function of the nervous system [119,120,121,122,123]. They are widely used worldwide because of their low price, broad spectrum of insecticidal efficiency, and ease of use. The excessive use of organochlorine pesticides will not only affect the environment but also cause harm to the human body [124,125,126]. Organochlorine pesticides mainly affect human health through food, respiration, and skin contact and can destroy certain hormones, enzymes, growth factors, and neurotransmitters in the body. Changes in relative homeostasis conditions within cells lead to oxidative stress and rapid cell death, leading to Parkinson’s [127], cancer [128], and endocrine and reproductive diseases.

**Figure 7:**
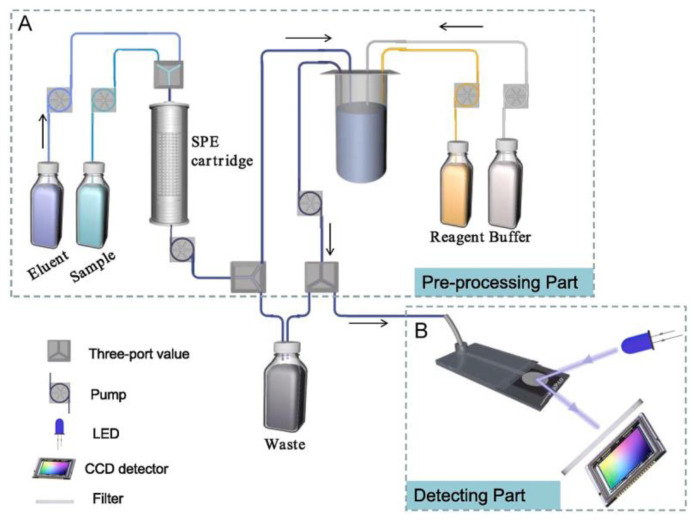
(**A**) Schematic diagram of the equipment. (**B**) Schematic diagram of fluorescence detection. (Reprinted/adapted with permission from Ref. [60]. Copyright 2022 *Food Chemistry*).

In order to detect organochlorine pesticide residues, many people have carried out research. Malik et al. successfully determined organochlorine pesticide residues using an electron capture detector (GC-ECD) in 2011 [129], and Chowdhury et al. achieved the same in 2013 using gas chromatography–tandem mass spectrometry (GC-MS) [130]. However, the testing equipment used requires professional personnel to operate it, and the equipment is expensive. In order to develop a simple, efficient, and stable method for the detection of organochlorine pesticide residues, Wang et al. developed a paper-based microfluidic device using fluorescence detection, as shown in Figure 7A [60], which consists of three three-port valves, six peristaltic pumps, and a 3D-printing-based, paper-based test platform (Figure 7B). The team proved the device’s practical applicability and high sensitivity with good recovery and close-to-peak detection of dicofol content in tea by adding multiple interference terms.

Pyrethroid pesticides are synthesized by simulating the chemical structure of natural pyrethroids, also known as biomimetic synthetic pesticides. They have a wide insecticidal spectrum, high efficacy, sterilization, and mold inhibition [131]. Pyrethroids have effectively reduced the incidence of malaria in Africa and other places [132], but overuse has seriously affected people’s health, causing cardiovascular diseases, reproductive diseases, and so on [133,134,135,136,137]. Pyrethroid analysis is routinely used in gas chromatography–electron capture detector (GC-ECD), gas chromatography–mass spectrometry (GC-MS), liquid chromatography–ultraviolet (LC-UV), and liquid chromatography–mass spectrometry (LC-MS). The instrument technologies mentioned above have high accuracy and precision, good sensitivity, and very low detection limits, but they are expensive, complex to operate, and unsuitable for most environments.

**Figure 8:**
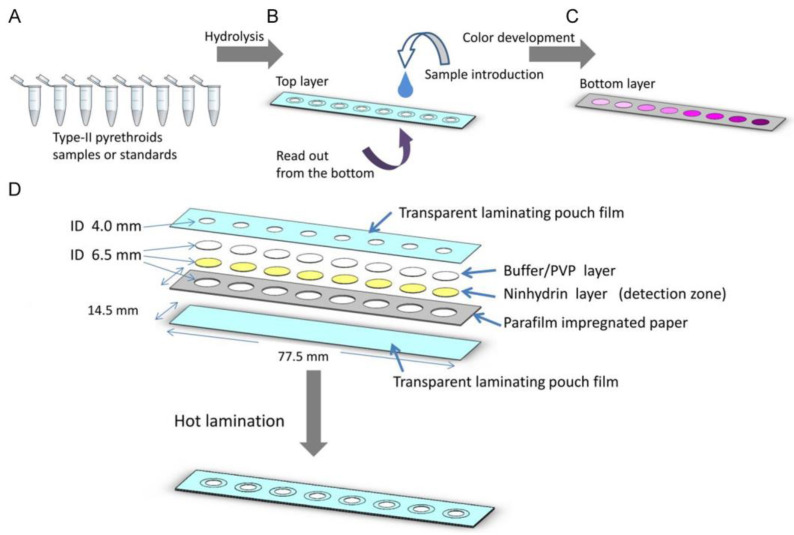
(**A**) Schematic diagram of pyrethroid hydrolysis. (**B**) Schematic diagram of the detection of paper-based microfluidic device. (**C**) Schematic diagram of color reaction with pyrethroid. (**D**) Design and dimensioning of the device. (Reprinted/adapted with permission from Ref. [61]. Copyright 2020 *Sensors*).

In order to develop a low-cost and convenient pyrethroid detection method [138], Sumate et al. developed a layered paper microfluidic device using colorimetric detection to screen pyrethroids type II [61], including cypermethrin, deltamethrin, cyhalothrin, and fenvalerate in environmental water samples. The detection principle is shown in Figure 8A,D; through cyanide ions and ninhydrin reaction color, on μPAD, a color intensity corresponding to pyrethroid pesticide concentration formed, using a red, green, and blue color-matching system for digital image analysis (Figure 8B,C). The detection limits for cypermethrin, deltamethrin, cyhalothrin, and fenvalerate were 2.50, 1.06, 3.20, and 5.73 μg/mL, respectively. Due to the paper-based layered structure, the device is easier to manufacture and use. It provides a detection platform for pesticide contamination in environmental surface water, with the advantages of portability, low reagent/sample consumption, and low-cost detection.

### 2.4. Commercialized Products

In order to meet the demand for quick and precise studies, several microfluidic devices have been created in the fields of food safety and pesticide detection. The commercial items that are currently utilized for pesticide testing are listed in Table 2 below. These gadgets use diverse microfluidic technology and have unique benefits and drawbacks.

For the quick and precise detection of pesticides in food and environmental samples, a number of microfluidic devices have been developed. The portable, highly sensitive My-coLab^TM^ AflaQuick^TM^ by EnviroLogix Inc. can identify aflatoxins in just 10 min. EnviroLogix Inc.’s Quick^TM^ has a high sensitivity and mobility level and can detect aflatoxins in under 10 min. Multiplexed pesticide detection is available with the Advanced Animal Diagnostics Raptor^TM^ Integrated Analysis Platform, although it is more expensive and demands specialist training. Pesticide identification is possible with the Biosensing Instrument Inc. ToxiQuant^TM^ Pesticide Microarray Kit. However, it requires refrigeration and has longer test times. The portable microfluidic sensor with SERS technology from GBC Scientific Equipment allows for label-free detection but calls for SERS equipment. The RapidChek^®^ SELECT^TM^ Salmonella from Romer Labs quickly identifies salmonella but has low sensitivity. Although it needs specialist equipment, Detection’s BioFlash Biological Identifier offers quick and sensitive findings. Mass spectrometry equipment is necessary for the ATHENA Integrated System, a lab-on-a-chip with quick results and customizable choices.

The microfluidic devices mentioned above have special features and capabilities to detect pesticides in food and environmental samples. While each technology has benefits like quick results, portability, and customizability choices, it also has drawbacks like con-strained detection targets, reduced sensitivity, the need for specialized equipment, and a range of prices. These aspects are important when choosing the best microfluidic device to meet the unique pesticide detection and food safety application demands.

## 3. Conclusions and Future Perspectives

Pesticide residues in food have an impact on human life and health. As more people become aware of food safety issues, researchers have started looking into several rapid, easy, and effective ways to check food safety, of which, microfluidic technology is one of the simplest and most effective methods. This paper reviews the latest developments in microfluidics for the detection of pesticide residues in food. Compared to traditional pesticide detection devices, microfluidic technology has the advantages of ease of use, low sample consumption, low reagent waste, and high sensitivity and accuracy. In particular, an increasing number of microfluidic detection technologies for pesticide residues have started to be integrated with 3D-μPAD, allowing for a greater variety of assay device de-signs and providing a direct and effective platform for pesticide detection in complex agricultural samples. In addition, a growing number of microfluidic devices are opting to use this combination of nanomaterials and microfluidic technology, as it allows for the enrichment of the target analyte between 10 and 100 times while using fewer reagents and obtaining better detection results and sensitivity.

Currently, there are three primary types of microfluidic technology detection methods for the detection of pesticide residues in food: colorimetric methods, fluorescence intensity methods, and electrochemical approaches. Each of these categories has its own benefits. Current research in fluorescence detection continues to focus on the monochromatic-fluorescence-based detection of pesticide residues, which has limited detection sensitivity. In the future, the contrast can be improved by adding a variety of colors to increase the detection sensitivity. Microfluidics is just beginning to develop in pesticide detection, but as people’s quality of life improves, they will become more concerned about food safety. With the development and exploration of 3D printing, nanomaterials and other technologies in the future, microfluidics will find more uses in food pesticide residue detection, providing simpler, more effective, fast, sensitive, and affordable methods.

## Figures and Tables

**Table 1:** Representative microfluidics devices for rapid detection of pesticide residues in food.

Pesticide Type	Detected Pesticides	Characteristics of the Microfluidic Devices and the Analysis	Sample	Ref.
Organophosphates	Paraoxon	LOD: 1 × 10^−4^ μg L^−1^ Assay time: 5 min QD-AChEaerogel-based microfluidic array sensor Linear range: 10^−12^–10^−5^ M	Apple	[24]
	Dichlorvos	LOD: 1.0 μg L^−1^ Assay time: 30 min Threaded 3D microfluidic paper analytical device-based sensor Linear range: 2.5–120 μg L^−1^	Spinach Tomato	[25]
	Malathion	LOD: 10.9 μg L^−1^ Assay time: 7 min A coulometric microdevice based on plug-based microfluidics Linear range: 10^−10^–10^−2^ M	Water	[56]
	Chlorpyrifos	LOD: 2.0 μg L^−1^ Assay time: 35 min Bioactive microfluidic paper device Linear range: 2.0–45 μg L^−1^	Water	[57]
Carbamate	Carbendazim	LOD: 3.102 μg L^−1^ Assay time: 12 min 3D-printed microfluidic nanosensors Linear range: 0.01–10 ppm	Cabbage	[23]
	Carbofuran	LOD: 199.1 μg L^−1^ Assay time: 6 min Electrochemical microfluidics based on carbon black nanoparticles Linear range: 25–125 μM	Water	[58]
	Carbaryl	LOD: 181.9 μg L^−1^ Assay time: 6 min A simple but robust droplet-based microfluidic system Linear range: 15.6–21.8 μM	Water	[59]
Organochlorine	Dicofol	LOD: 200 μg L^−1^ Assay time: 20 min A paper based microfluidic device modified via PTES Linear range: 0–10 ppm	Tea	[60]
Pyrethroid	Cypermethrin	LOD: 2500 μg L^−1^ Assay time: 6 min A microfluidic paper-based analytical device Linear range: 2–15 µg/mL	Water	[61]

**Table 2:** Commercialized products for pesticide testing.

Product Name	Manufacturer	Detected Pesticides	Types of Microfluidics Technology	Time	Target	Sensitivity	Storage and Stability	Ref.
MycoLab™ Afla-Quick™	EnviroLogix Inc.	Aflatoxins	Lateral Flow Immunoassay (LFIA)	10 min	Aflatoxins in various food samples	Detects aflatoxin B1, B2, G1, and G2 at low ppb levels	Stable at room temperature; shelf life of 12 months	[139]
Raptor™ Integrated Analysis Platform	Advanced Animal Diagnostics (AAD)	Various pesticides (customizable)	Microfluidic Immunoassay	30 min	Pesticides in food and feed samples	Customizable to different pesticide targets	Stable at room temperature; shelf life of 18 months	[140]
Pesticide Detect™	CerTest Biotec	Various pesticides (customizable)	Microfluidic Immunochromatography	10 min	Pesticides in food and water samples	Customizable to different pesticide targets	Stable at room temperature; shelf life of 12 months	[141]
ToxiQuant™ Pesticide Microarray Kit	Biosensing Instrument Inc.	Various pesticides (customizable)	Microarray-based detection	2–3 h	Pesticides in food and environmental samples	Customizable to different pesticide targets	Stable at refrigeration temperature; shelf life of 6 months	[142]
SERS-based Portable Microfluidic Sensor	GBC Scientific Equipment	Various pesticides (customizable)	Surface-Enhanced Raman Spectroscopy (SERS)	Minutes	Pesticides in food and water samples	Customizable to different pesticide targets	Stable at room temperature, dependent on the instrument	[143]
iTube	Ayanda Biosystems	Various pesticides (customizable)	Smartphone-based colorimetric assay	Minutes	Pesticides in food samples	Customizable to different pesticide targets	Stable at room temperature, dependent on the smartphone	[144]
RapidChek^®^ SELECT™ Salmonella	Romer Labs	Various pesticides (customizable)	Lateral Flow Immunoassay (LFIA)	15 min	Pesticides in food samples	Customizable to different pesticide targets	Stable at room temperature; shelf life of 12 months	[145]
BioFlash Biological Identifier	Smiths Detection	Various pesticides (customizable)	Immunomagnetic Separation (IMS)	Minutes	Pesticides in food and environmental samples	Customizable to different pesticide targets	Stable at room temperature, dependent on the instrument	[146]
MELISA-45 System	IBIS Technologies	Various pesticides (customizable)	Mass spectrometry	Minutes	Pesticides in food and environmental samples	Customizable to different pesticide targets	Stable at room temperature, dependent on the instrument	[147]
ATHENA Integrated System	Centre for Advanced BioNano Systems (Australia)	Various pesticides (customizable)	Lab-on-a-chip	Minutes	Pesticides in food samples	Customizable to different pesticide targets	Stable at room temperature, dependent on the instrument	[148]

## Data Availability

The data presented in this study are available upon request from the corresponding author.
